# Structural Differences between Human Proteins and Aero- and Microbial Allergens Define Allergenicity

**DOI:** 10.1371/journal.pone.0040552

**Published:** 2012-07-18

**Authors:** Helton da Costa Santiago, Sasisekhar Bennuru, José M. C. Ribeiro, Thomas B. Nutman

**Affiliations:** 1 The Laboratory of Parasitic Diseases, National Institute of Allergy and Infectious Diseases, National Institutes of Health, Bethesda, Maryland, United States of America; 2 Laboratory of Malaria and Vector Research, National Institute of Allergy and Infectious Diseases, National Institutes of Health, Rockville, Maryland, United States of America; Centre de Recherche Public de la Santé (CRP-Santé), Luxembourg

## Abstract

The current paradigm suggests that structural homology of allergenic proteins to microbial (particularly helminths) or human proteins underlie their allergenic nature. To examine systematically the structural relationships among allergens and proteins of pathogens (helminths, protozoans, fungi and bacteria) as they relate to allergenicity, we compared the amino acid sequence of 499 molecularly-defined allergens with the predicted proteomes of fifteen known pathogens, including Th2 inducing helminths and Th1-inducing protozoans, and humans using a variety of bioinformatic tools. Allergenicity was assessed based on IgE prevalences using publicly accessible databases and the literature. We found multiple homologues of common allergens among proteins of helminths, protozoans, fungi and humans, but not of bacteria. In contrast, 187 allergens showed no homology with any of the microbial genera studied. Interestingly, allergens without homologues or those with limited levels of sequence conservation were the most allergenic displaying high IgE prevalences in the allergic population. There was an inverse relationship between allergenicity and amino acid conservation levels with either parasite, including helminth, or human proteins. Our results suggest that allergenicity may be associated with the relative “uniqueness” of an antigen, i.e. immunogenicity, while similarity would lead to immunological tolerance.

## Introduction

There is a relative paucity of knowledge about what confers allergenicity [1], [2] to a given protein antigen. Nonetheless it has been observed that allergens are found within only 2% of all the known protein families [3] suggesting that not only host factors [4], [5] define the development of allergies, but also some intrinsic factors of the allergens themselves may be associated with their allergenic properties. One common feature that defines allergenicity is the ability to induce measurable levels of allergen-specific IgE. It is the cross-linking of this IgE bound to FcεR on the surface of mast cells and basophils that triggers the allergic reaction. However, the ability of a protein, or factor, to induce IgE is governed by the production of IL4, IL5 and IL13 (Th2 polarization) by T cells. Whether allergens per se have intrinsic properties required to polarize toward Th2-dominant response is still a matter of debate [6]. For example, some allergens, such as papain (an allergen of papaya, Car p 1) [7], and the helminth proteins, *S. mansoni* Omega 1 [8], [9] and a particular set of *S. mansoni* glycoproteins [10], [11], have been shown capable to cause Th2 polarization whereas for the majority of allergens or allergenic helminth proteins there is an extreme lack of information. Understanding the nature of allergenicity should provide new insights into therapy for and prevention of allergic diseases, conditions that are becoming increasingly more prevalent.

It has been observed that the increases in the prevalence of allergies throughout the world may be related to the increasingly cleaner environment and the relative availability of anti-microbials (e.g. antibiotics). Indeed the “Hygiene Hypothesis” suggests that the prevalence of allergic diseases is inversely associated with the prevalence of infectious diseases [12]. For example, certain viral [13], bacterial [14] and protozoan [15] infections are thought to be associated with protection from allergic diseases in both humans and experimental models. In addition, there is even more compelling evidence that helminth (worm) infections can also prevent the development of atopic diseases (reviewed in [16]). Interestingly, while viral, bacterial and protozoan infections can skew the immune response away from a Th2-dominated CD4+ T cell response – which underlies the development of allergy – acute (or early) helminth infection, in contrast, can favor Th2 responses with production of IL-4, IL-5 and IL-13 and large amounts of pro-allergenic IgE [17]. This early, type-2 dominated immune response [18], [19], [20] is likely to promote rather than attenuate atopy.

Immunological cross-reactivity among common allergens and helminth protein homologues has also been shown to contribute to the allergic sensitization that has been associated with acute helminth infection [21]. Indeed, there is clearly IgE cross-reactivity among helminth and aeroallergenic tropomyosins felt to reflect molecular and structural similarities. For example, IgE cross-reactivity has been demonstrated between helminth (e.g. filarial and Ascaris) tropomyosins and the tropomysoins of mites (Der p 10) [22], [23] and cockroaches (Bla g 7) [24]. In addition, cross-reactivity between cockroach glutathione-S transferase (GST; Bla g 5) and filarial GST of *Wuchereria bancrofti* has also been shown to occur [25].

The extensive number of homologues found between helminth proteins and allergens has led to the speculation that these similarities may underlie the allergenicity of IgE-inducing proteins [26]. This elegant hypothesis proposes that Th2-dominated immune responses have evolved to control helminth infection, but, because of molecular mimicry, the host also may become hyperresponsive to innocuous environmental proteins (allergens) leading to clinically apparent allergic disease [26]. This suggests that the “allergenicity” of some common allergens is a bystander effect that evolved as a consequence of natural immune responses to helminth antigens. Remarkably, structural homology is indeed involved in the allergenicity of Der p 2, a major house dust mite allergen that belongs to the same protein family of humans (MD2 protein) [27] and helminths (ML proteins).

The relative structural similarity of allergens with self-proteins have also been regarded as a factor underlying allergenicity [28], [29] either by interfering with specific host immune pathways [27] or by failing to induce “strong Th1 polarization” [29]. Although multiple mechanisms of allergic sensitization have been proposed [6], molecular mimicry – either with proteins of microbial pathogens (particularly by helminths) or of humans– seems to be a general unifying hypothesis to explain allergenicity [26], [28], [29].

Therefore, having systematic analyses of parasites and other microorganisms that could mimic (at a molecular level) allergens could allow for insight into potential mechanisms by which allergencity is conferred. To this end, we performed a comprehensive study of 499 molecularly-defined allergens and their structural relationship to predicted proteins of prototypical pathogens from which whole genome data were available so as to search for “allergen homologues” among the microbial genera. While our initial goal was to examine solely the helminth/allergy interface (e.g. *Brugia malayi*, *Loa loa*, *Wuchereria bancrofti* and *Schistosoma mansoni*), we broadened the analysis to include four protozoan, four bacterial and three fungal genomes in large part to understand the distinction between Th2-inducing organisms/proteins and those derived from organisms that more consistently induce Type-1 and Type-17 immune responses. This analysis enabled us to address if allergenicity is related to: a) structural similarities to helminth (Th2-inducing) or fungal (Th17-inducing) organisms that have many allergenic proteins; b) structural dissimilarities to proteins of protozoan or bacterial (Th1 inducing) organisms; or c) structural relatedness to human proteins. Our results suggest that immunogenicitybased on structural differences with human proteins is a major force driving the immune response to allergens.

## Results

### Extensive Molecular Similarity between Common Allergens and Parasite Proteins

We created a list of molecularly well-defined allergens covering 499 allergens catalogued in the AllFam database [3] that included 145/180 of the allergen families. This list was used to perform *in silico* searches for homologues across 15 pathogen genomes including four helminths (*Brugia malayi*, *Loa loa*, *Wuchereria bancrofti* and *Schistosoma mansoni*), four protozoans (*Leishmania major*, *Trypanosoma cruzi*, *Plasmodium falciparum* and *Toxoplasma gondii*), four bacteria (*Escherichia coli*, *Staphylococcus aureus*, *Mycobacterium tuberculosis* and *Listeria monocytogenis*) and three fungi (*Candida albicans*, *Histoplasma capsulatum* and *Aspergillus fumigatus*). A protein was considered homologous to the allergen if the expected value (e-value, that measures the likelihood of the amino acid match had occurred by chance) between them was less then 10^−6^, regardless the amino acid identity level. If the pair was not considered homologous (e-value equal to or greater than 10^−6^) the amino acid identity between them was arbitrarily set to zero.

**Figure 1:**
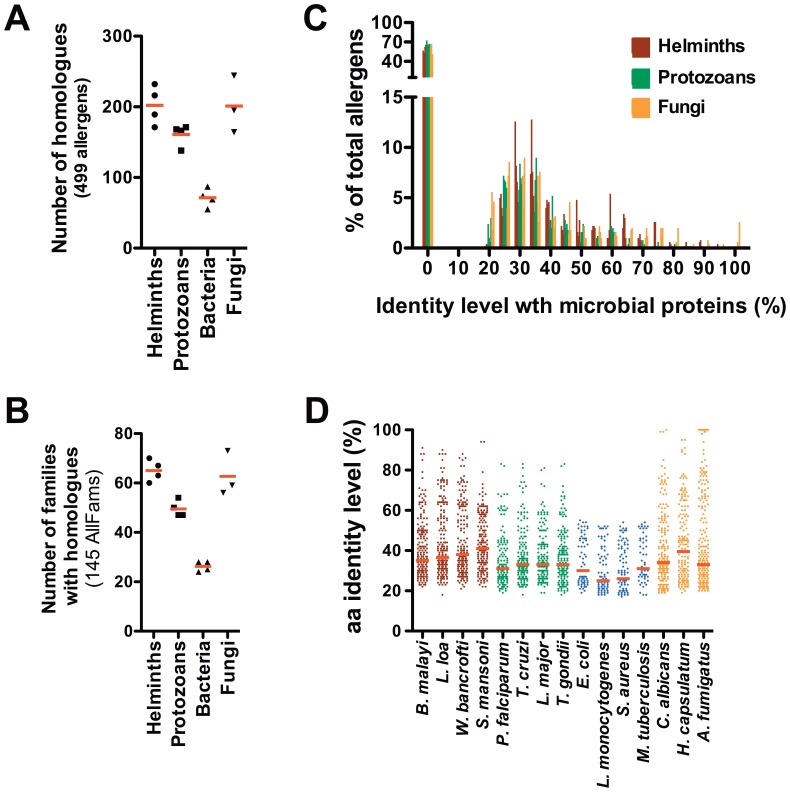
Allergens display large number of homologues in the genomes of helminths, protozoa and fungi. **A**, The number of homologues; **B**, the number of allergen families (AllFams) with homologues; and **C**, the frequency distribution of the homologues binned by the level of identity were assessed between allergens and the predicted proteomes of various microbial genera. Each dot (**A** and **B**) or spike (**C**) represents each microbe listed. **D**, Identity level between allergens and microbial pathogen proteins. Each dot represents an individual allergen with the identity level on the y-axis for a particular pathogen listed on the x-axis. Dots are colored coded to represent helminths in brown, protozoans in green, bacteria in blue and fungi in orange. The horizontal line represents the geometric mean for each pathogen listed.

We found a relatively large number of allergens having homologues among the many microbial organisms studied (Figure 1 and Table S1). Across the four helminth genera, the median number of encoded allergens with homologues was 202/499, representing 40% of the 499 allergens. This level of homology was similar to that found among the fungi studied (Figure 1A). Allergens had significantly fewer homologues (p<0.0001 by Fisher’s exact test) in protozoan genera (160/499; 32%) and even fewer in the bacteria group (71/499; 14%). This same pattern could be recognized in the number of allergen families (ALLFAM) involved (Figure 1B). Interestingly, if an allergen had a homologue in one genus of a certain phyla, it was also likely to have homologues in the other genera of the same phyla, with rare exceptions (Table S1 and Table S2). When there were homologues between the allergens and the proteins of the pathogenic organisms (except for the bacteria), for most of them (∼85% of the allergens) the amino acid identity levels were typically above 30% (Figure 1C). A majority of these showed an identity level between 25% and 45%. In contrast, the majority of the bacterial/allergen homologues had amino acid identity levels below 30% (Figure 1D). The median amino acid identity level between allergens and their respective homologous microbial proteins was 37% for helminths, 34% for fungi, 32% for protozoa and only 28% for bacteria (Figure 1D). We were thus able to confirm previous findings suggesting allergens are rarely homologous to bacterial proteins [30] probably because of big phylogenic distance. Because bacteria had fewer homologues and lower levels of homology when compared to the other microbes, this group was excluded from the subsequent analyses.

### Allergens Conserved with Microbial Proteins Display Lower Levels of Allergenicity

**Figure 2:**
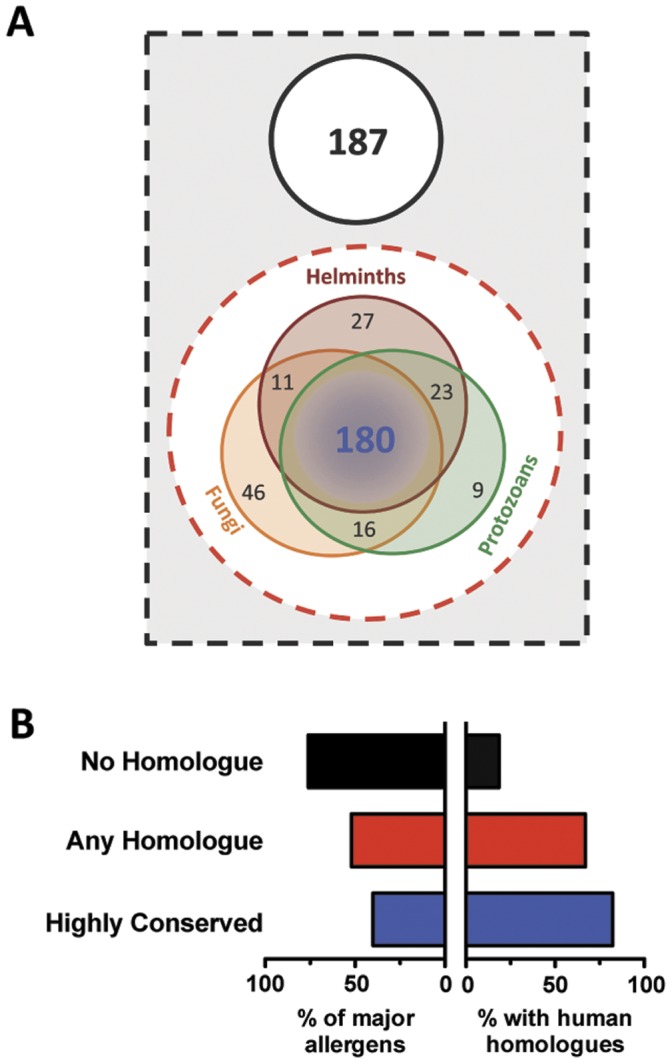
Unconserved allergens are more allergenic and less related to human proteins. **A**, Euler diagrams showing groups of allergens without (Black circle) or with homologues (red circle) among helminth (brown), fungi (orange) and protozoa (green). **B**, Allergens displaying no homologues (black), any homologues (red) or that were highly conserved (blue) in the parasitic phyla were categorized as major allergens (among 357 allergens for which information was available) (Left Panel) or by the presence of a human homologue (Right Panel).

**Figure 3:**
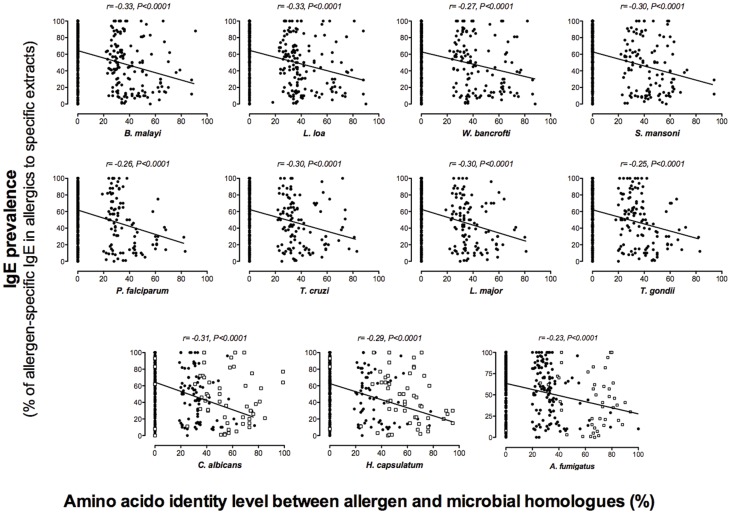
The level of allergenicity inversely correlates with the level of the conservation of the allergen. The IgE prevalence (y-axis) in a specific allergic population was plotted against the identity level (x-axis) between the allergen and its microbial homologue and correlation evaluated by Spearman rank test. Each dot represents one allergen and the lines represent the trend line. Allergens without homologues are stacked in the identity equal to zero area.

Among the 312 allergens displaying homologues in helminths, protozoa and fungi (the HPF group) of the 499 studied, we found 180 to be shared among the three organism groups analyzed (Figure 2A). In contrast, no homologues among any of the genera studied were found for 37% of the allergens (187/499) (Figure 2A) representing 43 of the 145 AllFams included. To evaluate the relationship between conservation of sequence and allergenicity, we collected the IgE prevalences for those allergens for which such information was available (Table S1) either from the literature or from the World Health Organization and the International Union of Immunology Societies (www.allergen.org) databases for systematic allergen nomenclature (a total of 357/499 allergens). We used the standard definition of a major allergen, that being molecularly defined substances from a complex mixture (extract) that induces IgE responses in >50% of patients allergic to that complex material source, i.e. allergen extract [31]. We found that 40% of those allergens that were highly conserved across the phyla were major allergens (blue bar of Figure 2B). In contrast, of the allergens that had no homologues in these 3 microbial phyla, 75% were major allergens (left black bar of Figure 2B) (p<0.0001 by Fisher’s exact test). These data suggest that there is a negative relationship between the level of conservation and the level of allergenicity. To address this observation further, we compared the existing IgE prevalence data for 357 known allergens with the identity level found in the respective homologue in each organism. Surprisingly, we found a consistent inverse relationship between allergenicity and the degree of identity between the allergen and its microbial homologue. The higher the identity level with a particular organisms’ homologue, the lower the IgE prevalence in the population (p<0.0001; r values varying from −0.23 and −0.33; Figure 3). In addition, the majority of those allergens with IgE prevalences above 50% showed either an identity level below 40% or none at all. In principle, there were no major allergens with identity levels above 80% except when the defined allergens were of helminth (i.e., Ani s 2) or fungal origin.

### Allergens with Homologues Among Parasites are Likely to Display Homologues in Human Genome

**Figure 4:**
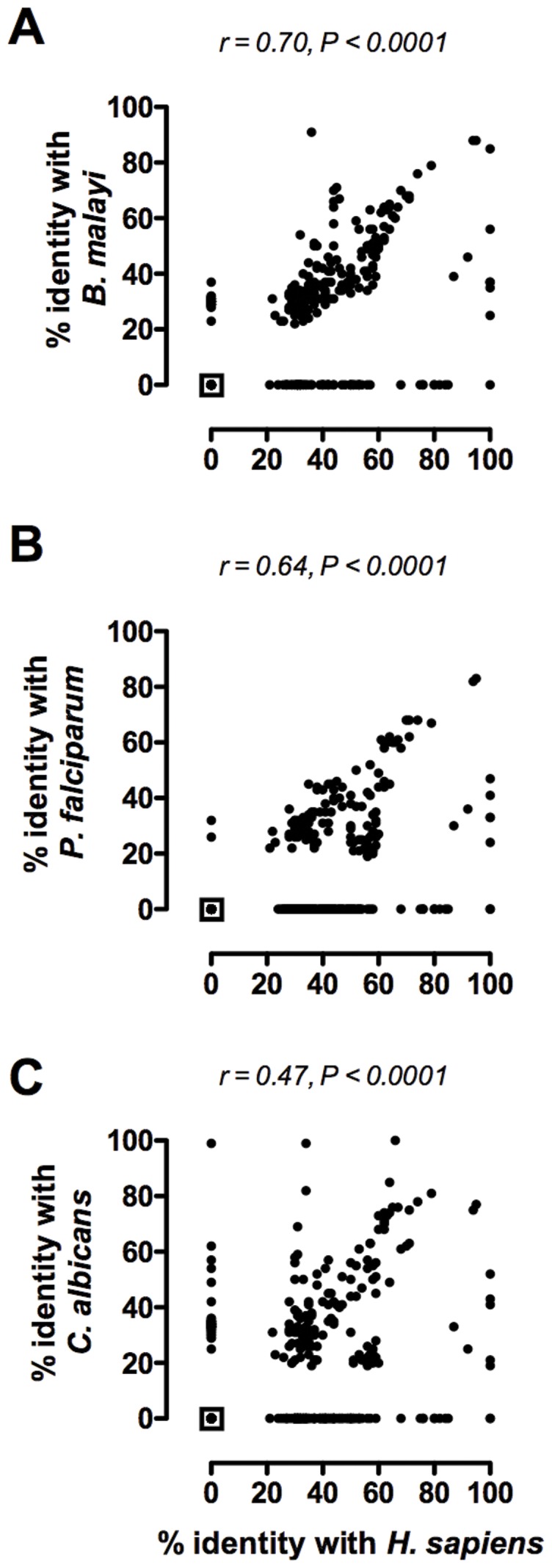
Allergens with homologues among microbes display similar levels of conservation with human proteins. The percent identity levels between human proteins (x-axis) a representative helminth (*B. malayi*, **A**), protozoan (*P. falciparum*, **B**) and fungus (*C. albicans*, **C**) protein (y-axis). Correlations were evaluated by Spearman rank test. Each dot represents one allergen and the squared-dot at the origin of the axis represents allergens that had neither homologues in humans nor in the microbes: (A) n = 222, (B) n = 247 and (C) n = 210 allergens.

**Figure 5:**
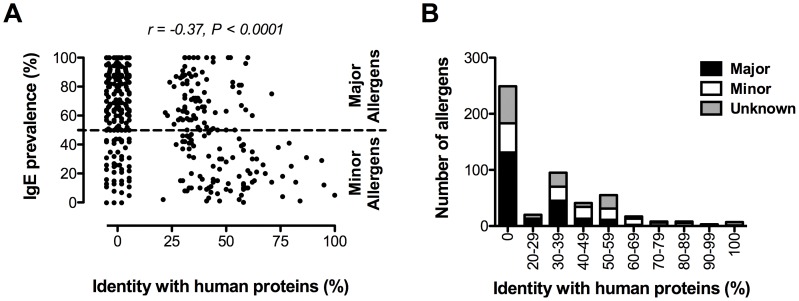
High levels of amino acid identity with human proteins are associated with decreased allergenicity. **A**, The prevalence of IgE to a natural purified or recombinant allergen (each dot represents a single allergen) and **B**, the absolute number of allergens (major, minor or unknown prevalence) as a function of the % amino acid identity with homologous human proteins (x-axis). Dotted line (A) separates major and minor allergens.

The finding that the prevalence of IgE to common allergens was inversely associated with the level of conservation with helminth and other organisms’ proteins prompted an examination of allergen/human protein conservation. Thus, we performed a similar search for homologues across the human genome. We found that half (250/499) of the allergens showed at least one homologue in humans; 86% of these (217/250) also had homologues in the HPF group. The percentage of human homologues increased from 18% for allergens without homologues in the HPF groups to 67% for those allergens that had homologues to any of the HPF group (Figure 2B). For the 180 allergens that showed homologues in the HPF, 82% also had homologues in the human genome suggesting that allergens conserved in HPF also were conserved in humans. To confirm this association, we analyzed the levels of amino acid identity between the allergens and a representative helminth (*B. malayi*), protozoan (*P. falciparum*) and fungal (*C. albicans*) organism as well as humans (Figure 4). We found highly significant positive correlations between the identity levels of allergens and the representative helminth (r = 0.70, P<0.0001), protozoan (r = 0.64, P<0.0001) and fungal (r = 0.47, P = 0.0011) organisms suggesting that a conserved protein allergen in HPF is also conserved to a similar level in human. Further analysis found a highly significant negative correlation between the prevalence of anti-allergen IgE and the amino acid sequence identity level with the human homologues (r = −0.37, P<0.0001) (Figure 5A). As observed between allergens and helminth proteins, the prevalence of allergen-specific IgE in the population is higher for allergens without human homologues than for those allergens with human homologues but with lower amino acid identities. Because the prevalence of IgE and their classification as major or minor allergens is based on population prevalences to specific extracts, this type of classification may induce a biased analysis if some major allergens are derived from proteins that are rarely allergenic as would be the case for Gal d 2 a major egg-white allergen that is rarely allergenic [32], [33], [34]. To avoid this possible bias, we performed correlation analyses based on the prevalence of IgE to 75 allergens in a population of over 23,000 patients with allergic symptoms [35]. We found a similar negative relationship using the prevalences reported (r = −0.37, P = 0.001). These data suggest that despite certain major allergens being of little importance in overall population, they contributed little to our analysis of 499 allergens.

Of note, the absolute number of allergens and their predicted allergenicity decreases dramatically when the identity level with human proteins is >40% (Figure 5B). Interestingly, there is only one major allergen with an identity level with its human homologue above 63%, Asp f 27 (a fungal cyclophilin). Indeed, major allergens that exceed the 40% human identity level threshold were only 10.8% (23/213) of the major allergens for which IgE prevalence data were available. In contrast, 27.7% (59/213) of the major allergens showed homologues with human proteins with identity level up to 40%, while 61.5% (131/213) of the major allergens had no human homologues. This observation not only shows that molecular conservation decreases the allergenicity of an environmental protein dramatically, but also demonstrates that even moderate similarity with self-protein appears to limit allergenicity.

Although including hundreds of allergens in our study strengthened the statistical analysis and the generalizability of our conclusions, it raised concern about the inclusion of several allergens from the same family. To avoid this bias, we performed correlation analysis using non-related allergens from specific extracts for which information could be obtained in Allergome and/or IUIS. We found similar negative correlation between conservation with human proteins and IgE prevalence for 13 non-duplicated allergens of *Dermatophagoides pteronyssinus* (Der p, r = −0.57, P = 0.040), 7 of *Phleum pratense* (Phl p, r = −0.80, P = 0.034), 16 allergens of *Aspergillus fumigatus* (Asp f, r = −0.64, P = 0.007) and a trend with the 9 non-duplicated allergens included of *Bos domesticus* (Bos d, r = −0.59, P = 0.097). Overall, for this specific group of allergens, the correlation was r = −0.58 (P<0.0001) (Table S3 and Figure S1).

## Discussion

In the present study, we challenge one of the major hypotheses used to explain the intrinsic property of a protein that define allergenicity, i.e. homology. Our results show that rather than structural similarity it is the differences among allergenic proteins and their human (or microbial) counterparts that drive allergenicity.

The development of allergies may be reflected not only by intrinsic host determinants, but also by factors related directly to the allergens themselves such as abundance within the allergenic extract, route of exposure to a given allergen, and the intrinsic, but yet undetermined, property that renders a protein allergenic. The most popular paradigm suggests that a protein’s similarity, either to proteins of microbial pathogens (i.e. Th2-inducing helminth parasites) or to humans, underlies their allergy-driving properties [26], [28], [29].

To address this hypothesis we compared 499 allergens with the whole deduced proteomes from the genomes of 15 microbes (including four helminths) and humans. We not only found an extraordinary level of conservation between allergens and helminth proteins, but we also found that the majority of allergens that had highly homologous proteins among the four helminth parasites also had homologues with protozoan, fungal and even human proteins. This finding indicates that similarity between allergens and helminth proteins does not underlie their allergenic properties, as intracellular protozoan parasites known to be major inducers of a Th1-dominated immune response [36] and fungi which are known to polarize toward the Th17-dominated response [36] have similar levels of homologous proteins to defined aero-allergens and at similar levels of amino acid identities.

Importantly, 37% of the defined allergens had no homologues to proteins from any of the organisms evaluated in this study; most of these “unique” allergens were found to be among the highly prevalent or, what is termed, major allergens. We found that the level of allergenicity of a given allergen decreased with increasing level of conservation to microbial proteins presumably because the level of conservation with the microbial proteins reflected that observed with human proteins. These finding agree with the current model of self-tolerance in which auto-reactive B and T lymphocytes are deleted during their development. Therefore, it is not surprising that allergens highly conserved with human proteins show the least degree of allergenicity. Interestingly, the inverse relationship between homology to human proteins and allergenicity has been suggested previously [37], but did not gain much traction with a single study showing that tropomyosins with identity levels greater than 55% with human tropomyosin were rarely allergenic [38].

Although the majority of major and highly prevalent allergens did not show homologues among the genomes analyzed, our results suggests that there is a selective window which was found to be between 30% and 40% amino acid identity with human (or helminth proteins) where the development of protein-specific IgE is less severely impaired. Above 40% identity with human proteins, the number of allergens and the level of allergenicity (as measured by anti-allergen IgE prevalence in a specific allergic population) decreased dramatically.

Interestingly, there are few major allergens with identities to human proteins above 50% (range 50–71%), most being calcium-binding proteins of fish (parvalbumins of the EF hand family), crustacea (tropomyosins) and fungi. Most of the environmental allergens showing a relative high level of identity with human proteins, however, were found to be minor allergens. Therefore, it is reasonable to speculate that for these groups structural differences *per se* may be sufficient to break immunologic self-tolerance and induce IgE optimally, or the presence of infection, such fungal infection, may drive a break of self-tolerance. Nevertheless, the general rule suggests that the failure of highly conserved allergens to induce IgE may reflect the deletion of T and B cells specific for cross-reacting self-proteins.

At this point, it is important to make a distinction between allergenicity and the ability to induce Th2 response. Our data clearly demonstrate that structural dissimilarity leads to increased allergenicity (defined by the induction of specific IgE responses). It may still be possible, however, that a non-human protein that is highly similar to a human protein may act as a T cell adjuvant, either by deviating the T cell response towards Th2 responses [28] or by failing to induce a strong Th1 response [29]. Therefore, it is reasonable to speculate that allergenicity favors the distance from the self while adjuvanticity may be associated with molecular mimicry. There may also be a “breakpoint”, so that an allergen may have characteristics of both strong antigenicity and strong adjuvanticity in which case 30–40% identity with self-proteins may be optimal.

Another important implication of the present study is related to cross-reactivity between allergens and parasite proteins. Having pre-formed allergen-specific IgE can have important implications for vaccine development and can lead potentially to serious allergic adverse events [39] following vaccination. If vaccinating individuals prior to the acquisition of the parasite (hookworm in this case [39]) could circumvent this problem, there would still be the concern of cross-reactive IgE to a homologous (non-helminth) allergen. For example, it has been demonstrated that Bla g 5 (a cockroach glutathione-S –transferase (GST) allergen) and helminth GST are cross-reactive in humans. Moreover, experimental helminth infection in mice can induce cross-sensitization to Bla g 5, a major cockroach allergen [25]. Although this phenomenon of cross-sensitization has yet to be formally demonstrated in humans, we presume that a significant proportion of new vaccines against infectious organisms might induce IgE-mediated responses because of pre-existing cross-reactive IgE to vaccine antigens. Indeed, epidemiological studies estimate the skin reactivity to cockroach extract to be ∼26% in North Americans and in people living in helminth-endemic areas [40]. Since the prevalence of anti-Bla g 5 IgE ranges between 30–90% of the cockroach allergic population, it suggests that at least 7% of a given population might be at risk for a cross-reactive allergy to a GST vaccine, two of which (schistosome-GST and hookworm GST) are being suggested as potential parasite candidates in humans [39], [41].

Finally, our results show that molecular uniqueness rather than molecular similarity between allergens and microbial/helminth or human proteins, underlies allergic responsiveness to environmental proteins, as close to a third of the allergens and allergen families, including most of the major allergens, fail to have homologous proteins among microbial genera. These results associate allergenicity with immunogenicity rather than with similarity. It is reasonable to assume that, for some allergens, similarity and functional mimicry play important roles in Th2 polarization [27] giving them properties of adjuvanticity. For the majority of allergens, however, similarity or relatedness to microbial and/or human proteins, leads to allergenic tolerance.

## Materials and Methods

### Allergen Lists and Genomes

A list of 1600 allergens were downloaded from the Allergome database (www. Allergome.org) and filtered for molecularly and immunologically defined allergens present in the Allergen Families (AllFam) [3] database (http://www.meduniwien.ac.at/allergens/allfam). After selection, a comprehensive list of 499 allergens, covering 145 of 180 AllFams listed, was used to build a fasta file containing the amino acid sequence of the allergens. This fasta file was used to perform *in silico* searches for homologues across 15 genomes of microbes and the human genome.

Fasta files with translated CDS annotated protein sequences for *Brugia malayi*, *Loa loa* and *Wuchereria bancrofti* (all version 1) were downloaded from the Broad Institute (http://www.broadinstitute.org/annotation/genome/filarial_worms/MultiDownloads.html), *Schistosoma mansoni* fasta file (version GeneDB v 4.0 h) was downloaded from Sanger Institute (ftp://ftp.sanger.ac.uk/pub/pathogens/), *Plasmodium falciparum* (PlasmoDB 6.4) from Plasmodb (http://plasmodb.org/common/downloads/), *Trypanosoma cruzi* CL Brener (TriTrypDB-2.2) and *Leishmania major* Friedlin (TriTrypDB-2.2) from Tritrypdb (http://tritrypdb.org/common/downloads/), *Toxoplasma gondii* ME-49 (ToxoDB 6.0) from Toxobd (http://toxodb.org/common/downloads/). Genome-predicted protein fasta file for *Escherichia coli* b088, *Listeria monocytogenes* EGD, *Staphyloccocus aureus* 55/2053, *Mycobacterium tuberculosis* C, *Aspergillus fumigatus*, *Candida albicans* WO-1 and *Histoplasma capsulatum* Nam1 were also downloaded from the Broad Institute, (http://www.broadinstitute.org//scientific-community/data). The *Homo sapiens* protein database were downloaded from NCBI genome website (ftp://ftp.ncbi.nlm.nih.gov/genomes/).

### Bioinformatics Tools and Analysis

The BlastP program [42] was used to compare the amino acid sequence of the list of allergens with each genome-predicted protein of the microbial/helminthic organisms and humans as previously described [43] Spreadsheets were generated containing the accession number of the protein-coding gene, E-value and percentage of identity at the amino acid level for the best homologue for each allergen along with IgE prevalences and hyperlinked literature references and can be found in Tables S1 and S2. Further analysis, statistics and graphs were performed using Excel (Microsoft Corporation) and Graphpad Prism v5.0 (GraphPad Software Inc., San Diego, California).

## Supporting Information

Figure S1 **Negative correlation between High levels of amino acid identity with human proteins was also observed for a group of non-redundant allergens.** The prevalence of IgE to a natural purified or recombinant allergen is represented in the y-axis and the level of identity with human proteins in the x-axis. Each dot represents a single allergen of the list showed in Table S3.
(TIF)

Table S1 **List of all allergens and the respective homologous proteins in the microbes and human genomes.**
(XLSX)

Table S2 **List of allergens families (ALLFam) analyzed and a representative allergen of each family.**
(DOCX)

Table S3 **Allergens used in Figure S1.**
(DOCX)
