# Supplementary figures and images for: Structural Differences between Human Proteins and Aero- and Microbial Allergens Define Allergenicity
Source: PLoS One. 2012 Jul 18;7(7):e40552. doi: 10.1371/journal.pone.0040552 (PMC3399830; doi:10.1371/journal.pone.0040552)

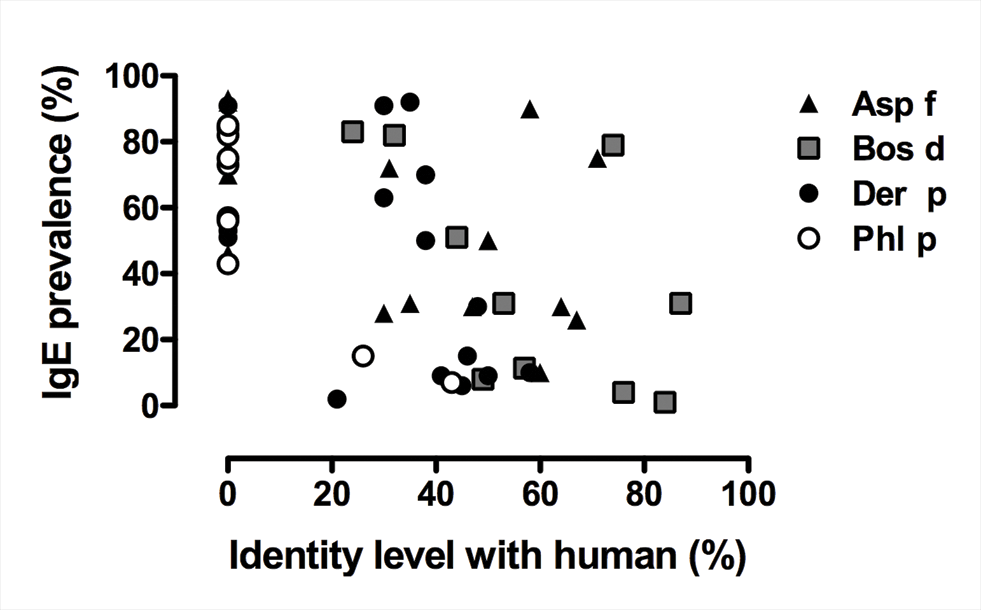

Supplement: Figure S1 — Negative correlation between High levels of amino acid identity with human proteins was also observed for a group of non-redundant allergens. The prevalence of IgE to a natural purified or recombinant allergen is represented in the y-axis and the level of identity with human proteins in the x-axis. Each dot represents a single allergen of the list showed in Table S3. (TIF) [file pone.0040552.s001.tif]
