# Supplementary material for: Structural Differences between Human Proteins and Aero- and Microbial Allergens Define Allergenicity
Source: PLoS One. 2012 Jul 18;7(7):e40552. doi: 10.1371/journal.pone.0040552 (PMC3399830; doi:10.1371/journal.pone.0040552)
Supplement: Table S2 — List of allergens families (ALLFam) analyzed and a representative allergen of each family. (DOCX) [file pone.0040552.s003.docx]

Table S2: List of allergen families (ALLFam) analyzed and a representative allergen of each family.

| **AllFam code** | **Family name** | **Representative Allergen** | **Allergen ACC#** | **Allergens evaluated^a^** | **Size of the family** | **Presence of homologue^b^** | | | | |
| --- | --- | --- | --- | --- | --- | --- | --- | --- | --- | --- |
|  |  |  |  |  |  | **Helminth** | **Protozoa** | **Fungus** | **Bacteria** | **Human** |
| AF001 | Helix-loop-helix DNA-binding domain | Fus c 3 | Q8J1X7 | 1 | 1 | X | X | X | O | X |
| AF002 | Heat shock protein Hsp70 | Alt a 3 | P78983 | 3 | 6 | X | X | X | X | X |
| AF003 | Animal Kunitz serine protease inhihibitor | Ani s 1 | Q7Z1K3 | 1 | 2 | X | O | O | O | X |
| AF004 | Eukaryotic aspartyl protease | Bla g 2 | P54958 | 1 | 10 | X | X | X | O | X |
| AF005 | Cystatin | Fel d 3 | Q8WNR9 | 1 | 4 | X | O | O | O | X |
| AF006 | Cytochrome c | Cur l 3 | Q96VP3 | 1 | 1 | X | X | X | O | X |
| AF007 | EF hand domain | Che a 3 | Q84V36 | 20 | 63 | X | X | X | O | X |
| AF008 | Intermediate filament protein | Hom s 5 | P02538 | 1 | 1 | X | X | X | X | X |
| AF009 | globin | Bos d Myoglobin | P02192 | 9 | 11 | O | O | O | O | X |
| AF010 | Glutathione S-transferase | Bla g 5 | O18598 | 2 | 8 | X | X | O | O | X |
| AF011 | Eukaryotic elongation factor 1 | Pen c 24 | Q69BZ7 | 1 | 1 | X | X | X | O | X |
| AF012 | Insulin family | Sus s Insulin | P01315 | 3 | 3 | O | O | O | O | X |
| AF013 | Kazal-type serine protease inhibitor | Gal d 1 | P01005 | 1 | 5 | X | X | O | O | X |
| AF014 | Lactate/malate dehydrogenase | Mala f 4 | Q9Y750 | 2 | 3 | X | X | X | X | X |
| AF015 | Lipocalin | Tyr p 13 | A7XZL4 | 12 | 23 | X | O | O | O | X |
| AF017 | Protein kinase | Sal k 2 | Q8L5K9 | 1 | 1 | X | X | X | X | X |
| AF018 | Serpin serine protease inhibitor | Gal d 2 | P01012 | 1 | 4 | X | X | O | O | X |
| AF019 | Cu/Zn Superoxide dismutase | Ole e 5 | P80740 | 1 | 1 | O | O | O | O | O |
| AF020 | Fe/Mn superoxide dismutase | Asp f 6 | Q92450 | 3 | 7 | X | X | X | X | X |
| AF021 | Subtilisin-like serine protease | Pen ch 18 | Q9P8G3 | 10 | 21 | X | X | X | X | X |
| AF023 | Thioredoxin | Asp f 28 | Q1RQJ1 | 3 | 11 | X | X | X | X | X |
| AF024 | Trypsin-like serine protease | Der p 3 | P39675 | 8 | 17 | X | O | O | O | X |
| AF025 | Tubulin/FtsZ family | Tyr p alpha Tubulin | Q52PV9 | 2 | 2 | X | X | X | O | X |
| AF026 | Pectin methylesterase | Sal k 1 | C1KET9 | 1 | 3 | X | O | X | O | O |
| AF027 | Cysteine-rich trypsin inhibitor-like domain | Api m 6 | Q27SJ7 | 1 | 3 | X | O | O | O | O |
| AF028 | Short-chain dehydrogenase | Alt a 8 | P0C0Y4 | 2 | 3 | X | X | X | X | X |
| AF029 | Zn-containing dehydrogenase | cand a 1 | P43067 | 1 | 1 | X | X | X | X | X |
| AF030 | Papain-like cysteine protease | Der p 1 | B5AYU7 | 8 | 10 | X | X | O | O | X |
| AF031 | Enolase | Alt a 6 | Q9HDT3 | 7 | 11 | X | X | X | X | X |
| AF032 | Triosephosphate isomerase | Tri a TPIS | Q9FS79 | 1 | 4 | X | X | X | X | X |
| AF033 | Alpha-amylase | Blo t 4 | A1KXI2 | 4 | 10 | O | O | X | O | X |
| AF034 | Legume lectin | Ara h Agglutinin | P02872 | 4 | 7 | O | O | O | O | O |
| AF035 | Peroxidase | Cap a 30kD | Q42661 | 3 | 5 | O | X | X | X | O |
| AF036 | Calcineurin-like phosphoesterase | Aed a 1 | P50635 | 1 | 2 | X | X | X | X | X |
| AF037 | Lipase | Pol d 1 | Q6Q252 | 8 | 11 | O | O | O | O | X |
| AF038 | Cyclophilin | Bet v 7 | P81531 | 2 | 5 | X | X | X | O | X |
| AF039 | Ribosome inactivating protein | Ade v RIP | Q70US9 | 7 | 8 | O | O | O | O | O |
| AF040 | Aldehyde dehydrogenase | Cla h 10 | P40108 | 2 | 3 | X | X | X | X | X |
| AF042 | Heat shock protein Hsp90 | Asp f 12 | P40292 | 1 | 1 | X | X | X | X | X |
| AF043 | Hevein-like domain | Hev b 11 | Q949H3 | 3 | 10 | O | X | X | O | O |
| AF044 | CRISP/PR-1/venom group 5 allergen family | Ves v 5 | Q05110 | 19 | 26 | X | O | X | O | X |
| AF045 | Cupin superfamily | Lup a 1 | B8Q5G0 | 15 | 42 | X | X | X | X | X |
| AF046 | Kunitz soybean trypsin inhibitor family | Sola t 2 | P16348 | 2 | 4 | O | O | O | O | O |
| AF047 | Catalase | Pen c 30 | Q2V6Q5 | 1 | 4 | O | X | X | X | X |
| AF048 | ATP synthase | Bos d OSCP | P13621 | 1 | 1 | X | X | X | X | X |
| AF049 | ATP:guanido phosphotransferase | Pen m 2 | Q8I9P7 | 5 | 9 | X | X | O | X | X |
| AF050 | Prolamin superfamily | Pis v 1 | B7P072 | 27 | 82 | X | O | O | O | O |
| AF051 | Profilin family | Ana c 1 | Q94JN2 | 33 | 48 | X | X | X | O | X |
| AF052 | Glycoside hydrolase family 32 | Lyc e 2 | Q8RVW4 | 1 | 3 | O | X | X | O | O |
| AF053 | Flavodoxin | Alt a 7 | P42058 | 2 | 2 | O | O | X | X | O |
| AF054 | Tropomyosin family | Blo t 10 | A7XZI8 | 26 | 47 | X | X | X | X | X |
| AF055 | Calreticulin family | Pen ch 31 | Q2TL59 | 1 | 1 | X | X | X | O | X |
| AF056 | Serum albumin | Bos d 6 | P02769 | 5 | 13 | O | O | O | O | X |
| AF057 | Polygalacturonase | Phl p 13 | Q9XG86 | 7 | 11 | O | O | X | O | O |
| AF058 | Ribosomal protein L3 | Asp f 23 | Q8NKF4 | 1 | 1 | X | X | X | O | X |
| AF059 | Art v 1 family | Art v 1 | Q84ZX5 | 1 | 2 | X | X | X | X | X |
| AF060 | Thaumatin-like protein | Mal d 2 | Q3BCT6 | 7 | 14 | O | O | O | O | O |
| AF061 | Prolyl oligopeptidase family | Ves v 3 | B1A4F7 | 2 | 5 | X | X | X | O | X |
| AF062 | Histidine acid phosphatase | Api m 3 | B6E2X9 | 1 | 2 | X | O | O | O | X |
| AF063 | Beta-1,3-glucanase | Ole e 9 | Q94G86 | 1 | 7 | O | X | O | O | O |
| AF064 | X8 domain | Ole e 10 | Q84V39 | 1 | 2 | O | O | O | O | O |
| AF065 | Alpha/beta casein | Bos d 8 alphaS1 | P02662 | 1 | 10 | O | O | O | O | X |
| AF066 | Haemocyanin | Per a 3 | Q25641 | 1 | 1 | O | O | O | O | O |
| AF068 | Transferrin | Gal d 3 | P02789 | 1 | 8 | X | O | O | O | X |
| AF069 | Bet v 1 -related family | Bet v 1 | Q42499 | 16 | 24 | O | O | O | O | O |
| AF070 | 60S acidic ribosomal protein | Asp f 8 | Q9UUZ6 | 6 | 10 | X | X | X | O | X |
| AF071 | Xylanase | Asp n Hemicellulase | P55329 | 1 | 1 | O | O | X | O | O |
| AF073 | Pectate Lyase | Pen c 32 | A2I7W3 | 10 | 10 | O | O | X | O | O |
| AF074 | Gelsolin family | Der f 16 | Q8MVU3 | 2 | 2 | X | O | X | O | X |
| AF075 | SGNH-hydrolase family | Hev b 4 | Q6T4P0 | 2 | 2 | O | O | O | O | O |
| AF076 | Glycoside hydrolase family 15 | Asp n Glucoamylase | P69328 | 1 | 1 | X | X | X | X | X |
| AF077 | Glycoside hydrolase family 18 | Der p 15 | Q4JK69 | 5 | 8 | X | X | X | X | X |
| AF078 | Chitin-binding peritrophin-A domain | Blo t 12 | Q17282 | 1 | 4 | O | O | O | O | O |
| AF079 | Glycoside hydrolase family 16 | Asp f 9 | B0XNL0 | 1 | 2 | X | X | X | X | X |
| AF080 | Glycoside hydrolase family 20 | Pen ch 20 | Q02352 | 1 | 1 | O | O |  | O | O |
| AF081 | GMC oxidoreductase | mala s 12 | Q5GMY3 | 1 | 1 | X | X | X | X | X |
| AF082 | Glyoxalase superfamily | Ory s Glyoxalase I | Q948T6 | 1 | 1 | X | X | X | X | X |
| AF083 | Glycoside hydrolase family 3 | Asp n 14 | O00089 | 1 | 2 | X | O | X | X | O |
| AF084 | Barwin family | Bra r 2 | P81729 | 1 | 2 | O | O | O | O | O |
| AF085 | Kappa-casein | Bos d 8 kappa | P02668 | 2 | 3 | O | O | O | O | X |
| AF086 | Staphylococcal/streptococcal toxin | Sta a SEB | P01552 | 6 | 8 | O | O | O | X | O |
| AF087 | Ole e 1-related protein | Ole e 1 | P19963 | 8 | 10 | O | O | O | O | O |
| AF088 | Casein kinase 2 regulatory subunit | Gal d Phosvitin | P67869 | 1 | 1 | X | X | X | O | X |
| AF089 | Lipid-binding serum glycoprotein | Equ c 4 | P82615 | 1 | 2 | O | O | O | O | X |
| AF090 | Oleosin | Cor a 13 | Q84T91 | 1 | 7 | O | O | O | O | O |
| AF091 | Diphtheria toxin | Cor d Toxoid | Q5PY51 | 1 | 1 | O | O | O | O | O |
| AF092 | Lipoprotein | Der p 14 | Q8N0N0 | 1 | 6 | X | X | O | O | X |
| AF093 | Expansin, C-terminal domain | Phl p 1 | P43213 | 15 | 22 | O | O | X | O | O |
| AF094 | Expansin, N-terminal domain | Hol l 1 | P43216 | 4 | 12 | O | O | X | O | O |
| AF095 | Melittin | Api m 4 | P01501 | 1 | 1 | O | O | O | O | O |
| AF096 | Beta-amylase | Hor v 17 | O23978 | 1 | 1 | O | O | O | O | O |
| AF097 | Collagen | Bos d alpha2I | P02465 | 1 | 4 | X | X | X | X | X |
| AF098 | Pheromone and odorant binding protein | Aed a 2 | P18153 | 1 | 4 | O | O | O | O | O |
| AF099 | Berberine bridge enzyme | Phl p 4 | Q2I6V7 | 6 | 7 | O | O | X | X | O |
| AF100 | Myosin tail | Der f 11 | Q967Z0 | 4 | 5 | X | X | X | X | X |
| AF102 | Group 5/6 grass pollen allergen | Dac g 5 | Q93WP9 | 8 | 9 | O |  | O | O | X |
| AF103 | Hyaluronidase | Dol m 2 | P49371 | 4 | 7 | X | O | O | O | X |
| AF104 | Patatin family | Hev b 7 | Q9SEM0 | 1 | 2 | X | X | X | O | O |
| AF105 | Clostridial neurotoxin | Clo t Toxoid | P04958 | 1 | 1 | O | O | O | O | O |
| AF106 | Class 3 Lipase | Rhi o Lipase | P61872 | 2 | 2 | X | X | X | O | O |
| AF107 | NAC domain | Hom s 2 | Q13765 | 1 | 1 | X | X | X | O | X |
| AF108 | DJ-1/PfpI family | Cand a 3 | Q6YK78 | 1 | 1 | O | O | X | O | O |
| AF109 | Fungalysin metalloprotease | Asp f 5 | P46075 | 1 | 1 | O | O | X | O | O |
| AF110 | Nuclear transport factor 2 | Alt a NTF2 | Q8NKB7 | 2 | 2 | X | X | X | O | X |
| AF111 | Mite Group 2 allergen family | Tyr p 2 | O02380 | 7 | 9 | O | O | O | O | X |
| AF112 | Plastocyanin-like protein | Amb a 3 | P00304 | 1 | 1 | O | O | O | O | O |
| AF113 | Ribonuclease N1 and T1 | Asp f 1 | P67875 | 1 | 2 | O | O | X | O | O |
| AF115 | High molecular weight glutenin | Tri a 26 | P10388 | 1 | 1 | X | X | X | X | X |
| AF116 | SART-1 family | Hom s 1 | O43290 | 1 | 1 | X | X | X | O | X |
| AF117 | Endonuclease/exonuclease/phosphatase family | Cim l Nitrophorin | O76745 | 1 | 1 | O | O | O | O |  |
| AF118 | Group 5 ragweed allergen | Amb a 5 | P02878 | 2 | 3 | O | O | O | O | O |
| AF119 | Triabin family | Bla g 4 | P54962 | 2 | 2 | O | O | O | O | O |
| AF121 | BCL7 family | Hom s 3 | Q9BQE9 | 1 | 1 | X |  | O | O | X |
| AF122 | Alliinase | All a Alliin lyase | P31756 | 3 | 3 | O | O | O | O | O |
| AF123 | Isoflavone reductase family | Cry j IFR | Q8RYC0 | 2 | 3 | O | O | X | O | O |
| AF124 | Apovitellenin I | Gal d Apo I | P02659 | 1 | 1 | O | O | O | O | O |
| AF125 | Rubber elongation factor family | Hev b 1 | P15252 | 1 | 2 | O | O | O | O | O |
| AF126 | Insect venom phospholipase A2 | Api m 1 | P00630 | 2 | 5 | O | O | O | O | X |
| AF127 | Group 1 cockroach allergen | Bla g 1 | O96522 | 2 | 2 | O | O | O | O | O |
| AF129 | Cerato-platanin | Asp f 15 | O60022 | 1 | 1 | O | O | X | O | O |
| AF131 | Redoxin | mal f 3 | P56578 | 6 | 6 | O | X | X | O | X |
| AF132 | Fibrinogen alpha-chain | Bos d Fibrin | P02672 | 1 | 1 | X | X | O | X | X |
| AF133 | Alginate lyase | Hev b Citrate BP | Q39962 | 1 | 1 | O | O | O | O | O |
| AF134 | Fel d 1 family | Fel d 1 | P30438 | 1 | 1 | O | O | O | O | O |
| AF135 | Ole e 6 family | Ole e 6 | O24172 | 1 | 1 | O | O | O | O | O |
| AF136 | Translationally controlled tumour protein | Cla h TCTP | A1KXP4 | 2 | 3 | X | X | X | O | X |
| AF137 | SXP/RAL-2 family | Ani s 8 | A7M6Q6 | 2 | 3 | X | O | O | O | O |
| AF138 | RuBisCO large chain | Spi o RuBisCO | P00870 | 1 | 1 | O | O | O | O | O |
| AF139 | Eukaryotic-type carbonic anhydrase | Dio o TSP | Q75N34 | 1 | 1 |  |  | O | X | X |
| AF140 | Carboxylesterase | Api m 8 | B2D0J5 | 1 | 2 | X | X | X | X | X |
| AF141 | Potato inhibitor I family | Tri a SPI | B3FHM6 | 1 | 2 | O | O | O | O | O |
| AF142 | WD-40 repeat | For t 2 | B2ZPG7 | 1 | 2 | X | X | X | O | X |
| AF143 | Leucine-rich repeat | Tri a 23kd | Q4W1G0 | 1 | 1 | O | X | O | X | O |
| AF145 | Phosphoglycerate kinase | Cand a PGK | P46273 | 1 | 1 | X | X | X | X | X |
| AF146 | Troponin I and T | Pon l 7 | P05547 | 1 | 1 | X | X | X | O | X |
| AF147 | Collagenase | Clo hi Collagenase | Q9S0X0 | 1 | 1 | O | O | O | O | O |
| AF148 | Snake venom phospholipase A2 | Bot as 1 | P24605 | 3 | 3 | X | O | O | O | X |
| AF149 | Proteasome subunit | Zea m 20S | Q5XML0 | 1 | 1 | X | X | X | O | X |
| AF150 | OmpA family | Hae in P6 | P10324 | 1 | 1 | O | O | O | X | O |
| AF151 | Hydrophobin | Cla h HCh1 | Q8NIN9 | 1 | 1 | O | O | O | O | O |
| AF152 | Cobalamin-independent methionine synthase | Sal k 3 | C1KEU0 | 1 | 1 | O | X | X | X | O |
| AF153 | Tick histamine binding protein | Arg r 1 | Q5GQ85 | 1 | 1 | O | O | O | O | O |
| AF154 | Major royal jelly protein | Api m Apalbumin 2 | O77061 | 2 | 2 | X | X | O | X | O |
| AF155 | GILT family | Tri a 27 | Q7Y1Z2 | 1 | 1 | X | X | O | O | X |
| AF156 | Group 5/21 mite allergen | Der p 5 | P14004 | 6 | 6 | O | O | O | O | O |
|  |  |  |  |  |  |  |  |  |  |  |
|  |  |  |  |  | TOTAL | 75 | 67 | 73 | 41 | 81 |

^a^Number of allergens evaluated in the respective family

^b^Red cells marked with X represent the presence of homologue and yellow cells marked with “O” represent their absence.
