# Supplementary material for: Structural Differences between Human Proteins and Aero- and Microbial Allergens Define Allergenicity
Source: PLoS One. 2012 Jul 18;7(7):e40552. doi: 10.1371/journal.pone.0040552 (PMC3399830; doi:10.1371/journal.pone.0040552)
Supplement: Table S3 — Allergens used in Figure S1. (DOCX) [file pone.0040552.s004.docx]

Table S3: A list of those allergens used in Figure S1

| **Allergen** | | **Identity w/ human (%)** | **Prevalence**  **(%)** | **Reference** | **IUSIS** | **ALLFAM code** | **ALLFAM** |
| --- | --- | --- | --- | --- | --- | --- | --- |
|  | |  |  |  |  |  |  |
| Asp f 1 | | 0 | 83 | [10848921](http://www.ncbi.nlm.nih.gov/pubmed/10848921?dopt=Abstract) | yes | AF113 | Ribonuclease N1 and T1 |
| Asp f 2 | | 0 | 100 | [9215251](http://www.ncbi.nlm.nih.gov/pubmed/9215251?dopt=Abstract) | yes | NA |  |
| Asp f 3 | | 31 | 72 | [9412580](http://www.ncbi.nlm.nih.gov/pubmed/9412580?dopt=AbstractPlus) | yes | AF131 | Redoxin |
| Asp f 4 | | 0 | 78 | [9482698](http://www.ncbi.nlm.nih.gov/pubmed/9482698?dopt=Abstract) | yes | NA |  |
| Asp f 5 | | 0 | 93 | [9482698](http://www.ncbi.nlm.nih.gov/pubmed/9482698?dopt=Abstract) | yes | AF109 | Fungalysin metalloprotease |
| Asp f 6 | | 50 | 56 | [9482698](http://www.ncbi.nlm.nih.gov/pubmed/9482698?dopt=Abstract) | yes | AF020 | Fe/Mn superoxide dismutase |
| Asp f 7 | | 0 | 46 | [9482698](http://www.ncbi.nlm.nih.gov/pubmed/9482698?dopt=Abstract) | yes | NA |  |
| Asp f 8 | | 60 | 10 | [10224291](http://www.ncbi.nlm.nih.gov/pubmed/10224291?dopt=Abstract) | yes | AF070 | 60S acidic ribosomal protein |
| Asp f 9 | | 35 | 31 | [9482698](http://www.ncbi.nlm.nih.gov/pubmed/9482698?dopt=Abstract) | yes | AF079 | Glycoside hydrolase family 16 |
| Asp f 10 | | 30 | 28 | [9482698](http://www.ncbi.nlm.nih.gov/pubmed/9482698?dopt=Abstract) | yes | AF004 | Eukaryotic aspartyl protease |
| Asp f 11 | | 58 | 90 | [11753999](http://www.ncbi.nlm.nih.gov/pubmed/11753999?dopt=Abstract) | yes | AF038 | Cyclophilin |
| Asp f 12 | | 65 |  |  | yes | AF042 | Heat shock protein Hsp90 |
| Asp f 13* | | 0 |  |  | yes | AF021 | Subtilisin-like serine protease |
| Asp f 15 | | 0 |  |  | yes | AF129 | Cerato-platanin |
| Asp f 16 | | 0 | 70 | [11422136](http://www.ncbi.nlm.nih.gov/pubmed/11422136?dopt=Abstract) | yes | AF079 | Glycoside hydrolase family 16 |
| Asp f 17 | |  |  |  | yes | AF181 | Hydrophobic surface binding protein A |
| Asp f 18 | | 31 | 79 | [11251631](http://www.ncbi.nlm.nih.gov/pubmed/11251631?dopt=AbstractPlus) | yes | AF021 | Subtilisin-like serine protease |
| Asp f 22 | | 64 | 30 | [11979043](http://www.ncbi.nlm.nih.gov/pubmed/11979043?dopt=AbstractPlus) | yes | AF031 | Enolase |
| Asp f 23 | | 67 | 26 | [12974759](http://www.ncbi.nlm.nih.gov/pubmed/12974759?dopt=Abstract) | yes | AF058 | Ribosomal protein L3 |
| Asp f 27* | | 71 | 75 | [16483252](http://www.ncbi.nlm.nih.gov/pubmed/16483252?dopt=AbstractPlus) | yes | AF038 | Cyclophilin |
| Asp f 28 | | 47 | 30 | [19032234](http://www.ncbi.nlm.nih.gov/pubmed/19032234?dopt=Abstract) | yes | AF023 | Thioredoxin |
| Asp f 29* | | 50 | 50 | [19032234](http://www.ncbi.nlm.nih.gov/pubmed/19032234?dopt=Abstract) | yes | AF023 | Thioredoxin |
| Asp f 34 | | 0 | 92 | [19416144](http://www.ncbi.nlm.nih.gov/pubmed/19416144?dopt=Abstract) | yes | NA |  |
|  | |  |  |  |  |  |  |
| Bos d 2 | | 24 | 83 | [18070162](http://www.ncbi.nlm.nih.gov/pubmed/18070162?dopt=Abstract) | yes | AF015 | Lipocalin |
| Bos d 3 | | 64 |  |  | yes | AF007 | EF hand |
| Bos d 4 | | 74 | 79 | [16606545](http://www.ncbi.nlm.nih.gov/pubmed/16606545?dopt=Abstract) | yes | AF016 | [C-type lysozyme/alpha-lactalbumin family](http://www.meduniwien.ac.at/allergens/allfam/factsheet.php?allfam_id=AF016) |
| Bos d 5* | | 44 | 51 | [20860558](http://www.ncbi.nlm.nih.gov/pubmed/20860558?dopt=Abstract) | yes | AF015 | Lipocalin |
| Bos d 6 | | 76 | 4 | [20860558](http://www.ncbi.nlm.nih.gov/pubmed/20860558?dopt=Abstract) | yes | AF056 | Serum albumin |
| Bos d 7 | |  | 35 | [8645993](http://www.ncbi.nlm.nih.gov/pubmed/8645993?dopt=Abstract) | yes | NA |  |
| Bos d 8 alphaS1 | | 32 | 82 | [19454699](http://www.ncbi.nlm.nih.gov/pubmed/19454699?dopt=Abstract) | yes | AF065 | Alpha/beta casein |
| Bos d 8 kappa | | 53 | 31 | [18307527](http://www.ncbi.nlm.nih.gov/pubmed/18307527?dopt=Abstract) | yes | AF085 | Kappa-casein |
| Bos d alpha2I | | 92 | rare | [12373276](http://www.ncbi.nlm.nih.gov/pubmed/12373276?dopt=Abstract) | yes | AF097 | Collagen |
| Bos d Chymosin | | 57 | 11.4 |  | no | AF004 | Eukaryotic aspartyl protease |
| Bos d Fibrin | | 59 |  | [8721528](http://www.ncbi.nlm.nih.gov/pubmed/8721528?dopt=Abstract) | no | AF132 | Fibrinogen alpha-chain |
| Bos d Insulin | | 80 |  |  | no | AF012 | Insulin family |
| Bos d Myoglobin | | 84 | 1 | [14982516](http://www.ncbi.nlm.nih.gov/pubmed/14982516?dopt=Abstract) | no | AF009 | globin |
| Bos d OSCP | | 87 | 31 | [21136777](http://www.ncbi.nlm.nih.gov/pubmed/21136777) | no | AF048 | ATP synthase |
| Bos thrombin | | 81 | 28 | [14656362](http://www.ncbi.nlm.nih.gov/pubmed/14656362?dopt=Abstract) | no | AF024 | [Trypsin-like serine protease](http://www.meduniwien.ac.at/allergens/allfam/factsheet.php?allfam_id=AF024) |
| Bos d TI | | 49 | 8 | [9576225](http://www.ncbi.nlm.nih.gov/pubmed/9576225?dopt=Abstract) | no | AF003 | [Animal Kunitz serine protease inhihibitor](http://www.meduniwien.ac.at/allergens/allfam/factsheet.php?allfam_id=AF003) |
|  | |  |  |  |  |  |  |
| Der p 1 | | 30 | 91 | [19021722](http://www.ncbi.nlm.nih.gov/pubmed/19021722?dopt=Abstract) | yes | AF030 | Papain-like cysteine protease |
| Der p 2 | | 0 | 91 | [19021722](http://www.ncbi.nlm.nih.gov/pubmed/19021722?dopt=Abstract) | yes | AF111 | Mite Group 2 allergen family |
| Der p 3 | | 41 | 9 | [16890759](http://www.ncbi.nlm.nih.gov/pubmed/16890759?dopt=Abstract) | yes | AF024 | Trypsin-like serine protease |
| Der p 4 | | 48 | 30 | [19021722](http://www.ncbi.nlm.nih.gov/pubmed/19021722?dopt=Abstract) | yes | AF033 | Alpha-amylase |
| Der p 5 | | 0 | 51 | [19021722](http://www.ncbi.nlm.nih.gov/pubmed/19021722?dopt=Abstract) | yes | AF156 | Group 5/21 mite allergen |
| Der p 7 | | 0 | 53 | [10779281](http://www.ncbi.nlm.nih.gov/pubmed/10779281?dopt=AbstractPlus) | yes | NA |  |
| Der p 8 | | 50 | 9 | [19021722](http://www.ncbi.nlm.nih.gov/pubmed/19021722?dopt=Abstract) | yes | AF010 | Glutathione S-transferase |
| Der p 9* | | 35 | 92 | [8876548](http://www.ncbi.nlm.nih.gov/pubmed/8876548?dopt=AbstractPlus) | yes | AF024 | Trypsin-like serine protease |
| Der p 10 | | 58 | 10 | [19021722](http://www.ncbi.nlm.nih.gov/pubmed/19021722?dopt=Abstract) | yes | AF054 | Tropomyosin family |
| Der p 11 | | 38 | 50 | [15005727](http://www.ncbi.nlm.nih.gov/pubmed/15005727?dopt=AbstractPlus) | yes | AF100 | Myosin tail |
| Der p 14 | | 21 | 2 | [19021722](http://www.ncbi.nlm.nih.gov/pubmed/19021722?dopt=Abstract) | yes | AF092 | Lipoprotein |
| Der p 15 | | 38 | 70 | [16776685](http://www.ncbi.nlm.nih.gov/pubmed/16776685?dopt=Abstract) | yes | AF077 | Glycoside hydrolase family 18 |
| Der p 18* | | 30 | 63 | [16776685](http://www.ncbi.nlm.nih.gov/pubmed/16776685?dopt=Abstract) | yes | AF077 | Glycoside hydrolase family 18 |
| Der p 20 | | 46 | 15 | [16890759](http://www.ncbi.nlm.nih.gov/pubmed/16890759?dopt=Abstract) | yes | AF049 | ATP:guanido phosphotransferase |
| Der p 21 | | 0 | 56 | [18445190](http://www.ncbi.nlm.nih.gov/pubmed/18445190?dopt=Abstract) | yes | AF156 | Group 5/21 mite allergen |
| Der p 23* | | 45 | 6 | [21332797](http://www.ncbi.nlm.nih.gov/pubmed/21332797?dopt=Abstract) | yes | AF015 | [Lipocalin](http://www.meduniwien.ac.at/allergens/allfam/factsheet.php?allfam_id=AF015) |
|  | |  |  |  |  |  |  |
| Phl p 1 | | 0 | 84 | [18339006](http://www.ncbi.nlm.nih.gov/pubmed/18339006?dopt=Abstract) | yes | AF093 | Expansin, C-terminal domain |
| Phl p 2* | | 0 | 82 | [18339006](http://www.ncbi.nlm.nih.gov/pubmed/18339006?dopt=Abstract) | yes | AF093 | Expansin, C-terminal domain |
| Phl p 3* | | 0 | 57 | [16776686](http://www.ncbi.nlm.nih.gov/pubmed/16776686?dopt=Abstract) | no | AF093 | Expansin, C-terminal domain |
| Phl p 4 | | 0 | 85 | [18339006](http://www.ncbi.nlm.nih.gov/pubmed/18339006?dopt=Abstract) | yes | AF099 | Berberine bridge enzyme |
| Phl p 5* | | 0 | 73 | [18339006](http://www.ncbi.nlm.nih.gov/pubmed/18339006?dopt=Abstract) | yes | AF102 | Group 5/6 grass pollen allergen |
| Phl p 6 | | 0 | 75 | [10553075](http://www.ncbi.nlm.nih.gov/pubmed/10553075?dopt=AbstractPlus) | yes | AF102 | Group 5/6 grass pollen allergen |
| Phl p 7 | | 43 | 7 | [12534548](http://www.ncbi.nlm.nih.gov/pubmed/12534548?dopt=Abstract) | yes | AF007 | EF hand domain |
| Phl p 11 | | 0 | 43 | [12534548](http://www.ncbi.nlm.nih.gov/pubmed/12534548?dopt=Abstract) | yes | AF087 | Ole e 1-related protein |
| Phl p 12 | | 26 | 15 | [12534548](http://www.ncbi.nlm.nih.gov/pubmed/12534548?dopt=Abstract) | yes | AF051 | Profilin family |
| Phl p 13 | | 0 | 56 | [18339006](http://www.ncbi.nlm.nih.gov/pubmed/18339006?dopt=Abstract) | yes | AF057 | [Polygalacturonase](http://www.meduniwien.ac.at/allergens/allfam/factsheet.php?allfam_id=AF057) |
| Allergens marked with asterisk were not included in the analysis to avoid duplication of families. | | | | | | |  |
